# Critical Intestinal Perforations in Pediatric Immunocompromised Patients: A Case-Based Review

**DOI:** 10.3390/pediatric18010030

**Published:** 2026-02-14

**Authors:** William Hunt Stafford, Jennifer McArthur, Saad Ghafoor

**Affiliations:** 1 Division of Pediatric Critical Care, University of Tennessee Health Science Center, Memphis, TN 38103, USA; 2 Critical Care Medicine Division, St. Jude Children’s Research Hospital, Memphis, TN 38105, USA; jennifer.mcarthur@stjude.org

**Keywords:** intestinal perforation, immunocompromised host, pediatric oncology, pediatric intensive care, hematopoietic stem cell transplant, hemophagocytic lymphohistiocytosis

## Abstract

As survival rates for children with cancer and immune disorders have improved, clinical focus has shifted toward managing serious treatment-related complications. Intestinal perforation remains life-threatening and is typically diagnosed by signs of peritonitis and inflammation. This report presents three high-risk pediatric patients who developed severe intestinal perforation without the usual clinical symptoms. Each patient was receiving high-dose corticosteroids and/or targeted biologic immunomodulators (ruxolitinib, anakinra, tocilizumab, eculizumab). Classic indicators such as fever, leukocytosis, hemodynamic instability, and abdominal pain were absent, despite surgical findings of fecal contamination and bowel necrosis. All three patients survived to hospital discharge. These cases demonstrate that potent immunomodulatory therapies can mask the physiological response to perforation. Relying solely on traditional clinical signs may delay diagnosis. In this population, subtle findings such as persistent gastrointestinal bleeding, feeding intolerance, or minor imaging abnormalities should prompt consideration of perforation. Early imaging and multidisciplinary review are essential for timely intervention and improved outcomes.

## 1. Introduction

Since the mid-1970s, five-year survival rates for children and adolescents with cancer have risen from approximately 50–60% to 80–90% [1]. This improvement is attributable to better diagnostic tests, targeted chemotherapy, improved risk assessment, enhanced supportive care, and advances in surgery [2,3]. As more patients survive, the focus has shifted from preventing death to also reducing treatment-related complications.

Intestinal perforation is a rare but serious complication in children and adolescents receiving treatment for leukemia or other immune disorders. Its etiology remains poorly understood, despite a strong association with significant morbidity and mortality. Two main mechanisms have been identified: (1) spontaneous perforation of primary gastrointestinal tumors or perforation following rapid chemotherapy-induced tumor necrosis, and (2) progression of neutropenic enterocolitis due to myelotoxic therapy, leading to ischemia, necrosis, and perforation [4,5,6]. Additionally, these patients often receive immunosuppressive therapies, including corticosteroids, which cause lymphoid depletion within Peyer’s patches. This is believed to impair gastrointestinal immune defenses, decrease peristalsis, and weaken bowel wall integrity [7]. Hematopoietic stem cell transplantation (HSCT) increases the risk of gastrointestinal complications, particularly graft-versus-host disease (GVHD) and thrombotic microangiopathy (TMA), which can damage the intestines by injuring small blood vessels and reducing blood flow [8,9].

Patients with intestinal perforation continue to face high rates of illness and death, with mortality reported at 40–50% [5]. Diagnosis usually relies on classic signs of peritonitis, but with increased use of advanced immunomodulatory drugs, these signs may be absent. There is a reported association and a potentially increased risk of gastrointestinal perforation associated with the use of biologic agents such as tocilizumab, ruxolitinib, and rituximab in adults [10,11,12,13]. Nevertheless, the mechanisms underlying this potential risk remain poorly understood. Furthermore, limited published research exists regarding silent or asymptomatic intestinal perforation in the era of targeted biologic immunosuppression. This case series presents three high-risk pediatric patients with intestinal perforation who lacked the typical warning signs prior to diagnosis.

## 2. Cases

### 2.1. Case Identification and Selection

We report three cases of silent intestinal perforation admitted to our institution’s intensive care unit from 2024 to 2025. This constitutes a retrospective descriptive case series without a prospective screening component.

#### 2.1.1. Case 1

A 15-year-old male with relapsed acute myeloid leukemia was admitted after a syncopal episode. His course was complicated by multidrug-resistant Escherichia coli sepsis, severe neutropenic enterocolitis, acute respiratory failure, cardiomyopathy, and GVHD. Immunosuppression at admission included a steroid taper and ruxolitinib. On admission, he was tachycardic and hypotensive. Laboratory tests showed hemoglobin of 4.5 g/dL (down from 7.6 g/dL the day before), leukopenia without neutropenia, thrombocytopenia, and mild hyponatremia. A contrast-enhanced CT of the abdomen and pelvis showed thickening of the ascending colon with surrounding fat stranding, unchanged from prior imaging and attributed to previous neutropenic enterocolitis. He was started on bowel rest, IV fluids, and levofloxacin.

**Figure 1:**
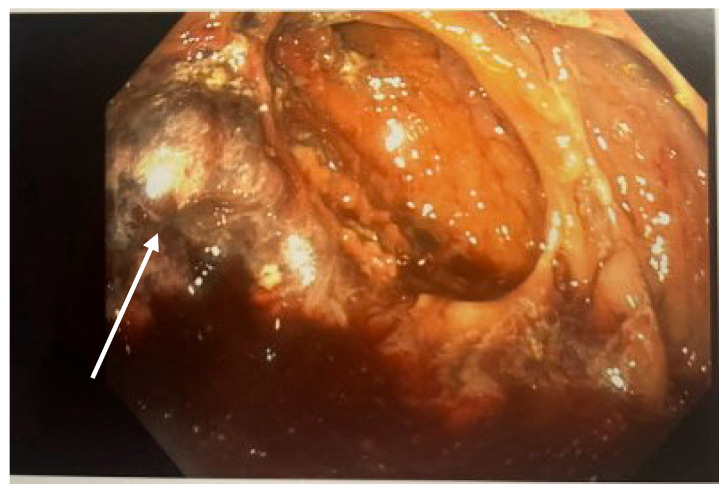
Photo from colonoscopy showing necrotic intestinal tissue (white arrow).

**Figure 2:**
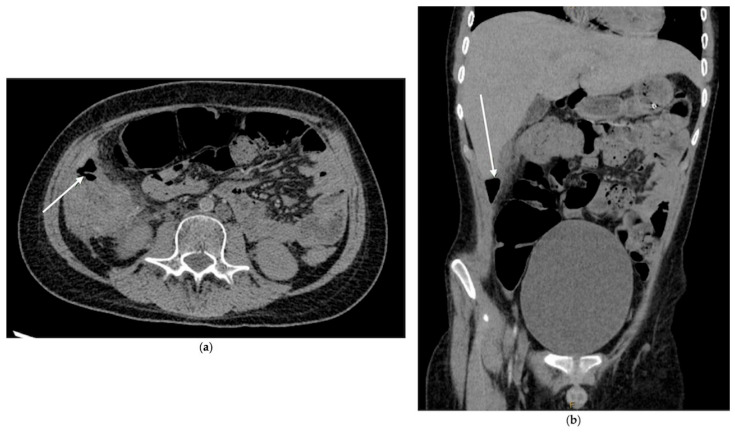
CT (**a**) axial image and (**b**) coronal image with white arrows indicating free air within the right upper quadrant.

In the week before diagnosis, he had progressively worsening hematochezia but remained hemodynamically stable without fever, tachycardia, or hypotension. Serial abdominal exams were consistently soft, non-distended, and non-tender. Inflammatory markers were not elevated. A colonoscopy for ongoing bleeding revealed maroon stool and necrotic tissue in the cecum, as seen in Figure 1. A re-review of the admission CT scan identified a previously missed small pocket of free air anterior to the ascending colon, seen in Figure 2. Immediately pre-operatively, he was afebrile, in no distress, with a soft abdomen. Labs showed a normal white blood cell count and no metabolic acidosis.

He underwent an urgent exploratory laparotomy, which revealed a rupture of the hepatic flexure into the retroperitoneum with fecal contamination. A right hemicolectomy was performed. His postoperative course was complicated by refractory septic shock and multiorgan dysfunction due to resistant *E. coli* bacteremia. He gradually recovered, was extubated, and transferred out of the ICU. He survived to hospital discharge.

#### 2.1.2. Case 2

A four-year-old girl with acute lymphoblastic leukemia underwent a haploidentical hematopoietic stem cell transplant. On day thirteen post-transplant, she developed acute liver failure, shock, multiorgan dysfunction, pneumatosis intestinalis, and coagulopathy with thrombotic microangiopathy (TMA). She was treated with defibrotide and eculizumab, with improvement. Immunosuppression at the time of perforation included eculizumab, methylprednisolone, ruxolitinib, defibrotide, and hydrocortisone. Antibiotic coverage was piperacillin-tazobactam.

On day 37 post-transplant, her peritoneal drain output appeared fecal. Prior to this, her vital signs were stable without fever or tachycardia. Her abdomen had been distended and firm with decreased bowel sounds for several days. She tolerated small feedings but had no bowel movements. Laboratory trends were not concerning.

**Figure 3:**
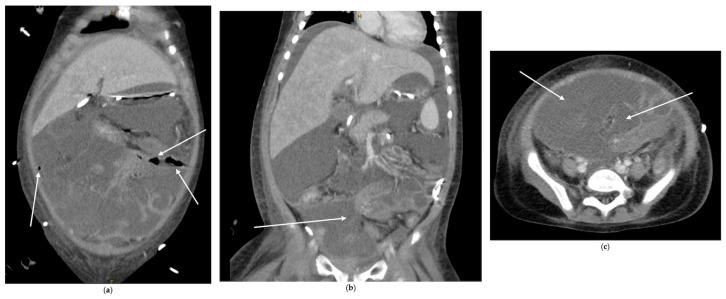
CT images: (**a**) coronal image showing pockets of free air throughout abdomen; (**b**) coronal image and (**c**) axial image showing complex fluid collection in the lower abdomen and pelvis. Areas highlighted by white arrows.

A CT scan showed loculated ascites, free intra-abdominal air, and colonic wall thickening, as seen in Figure 3. She underwent urgent surgery, which revealed severe fecal peritonitis and gangrenous small bowel with multiple perforations. Sixty centimeters of small bowel were resected, and an ostomy was created. Postoperatively, she developed adenovirus pneumonia requiring prolonged ventilation but was eventually extubated and transferred from the ICU. She survived to hospital discharge.

#### 2.1.3. Case 3

A three-year-old girl, born at 32 weeks’ gestation, was transferred to the ICU with fever, decreased oral intake, and oliguria. Evaluation revealed markedly elevated inflammatory markers and hepatosplenomegaly, leading to a diagnosis of secondary hemophagocytic lymphohistiocytosis (HLH) triggered by Epstein–Barr virus. She was treated with anakinra, dexamethasone, and rituximab. Later, ruxolitinib and tocilizumab were added for persistent inflammation.

**Figure 4:**
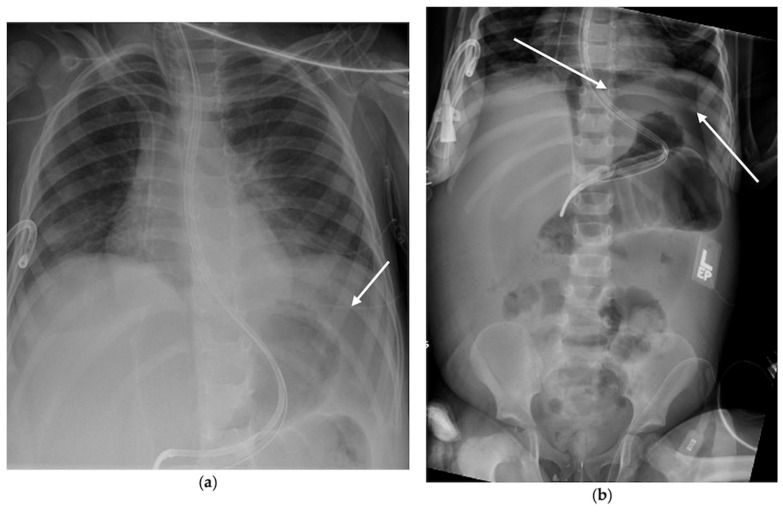
(**a**) AP x-ray of chest showing lucency beneath the left hemidiaphragm concerning for free air; (**b**) Confirmatory AP x-ray of abdomen showing free air beneath the left hemidiaphragm. Areas highlighted by white arrows.

She required intubation early in her course but improved with therapy and was extubated on hospital day eight. The following morning, a routine chest radiograph showed free intraperitoneal air, seen in Figure 4. Prior to this finding, she was clinically improving, with stable hemodynamics, no changes on abdominal exam, normalization of inflammatory markers, and tolerance of enteral feeds. Exploratory laparotomy revealed gross ascites, necrotic distal ileum and cecum, and an ileal perforation. She underwent ileocecectomy and a diverting loop ileostomy.

A retrospective review of prior chest radiographs showed subtle free air for several days before clinical recognition. Her postoperative course was prolonged, yet she survived.

## 3. Discussion

### 3.1. The Evolving Challenge of Clinically Silent Catastrophes in High-Risk Pediatric Care

The substantial improvement in survival rates for children with cancer and immune disorders is a significant medical achievement [1]. However, clinical focus has increasingly shifted toward reducing serious treatment-related complications [2,3]. Intestinal perforation remains a major risk, with mortality of 40–50% even when identified early [5,6]. The three cases described herein challenge conventional diagnostic approaches, which rely on classic symptoms such as acute abdominal pain, guarding, fever, and inflammatory signs [4,5,6,14,15]. Each patient had severe intestinal perforation with fecal contamination and bowel necrosis but lacked the expected clinical warning signs. This series highlights a critical and often overlooked issue: silent intestinal perforation in immunocompromised children receiving modern immunosuppressive therapies, necessitating a reassessment of risk evaluation and monitoring strategies for this complication.

### 3.2. Blunted Inflammatory Signaling in Injury: Exploring the Role of Advanced Immunomodulation

All three patients experienced profound immunosuppression because of their therapeutic regimens, beyond the effects of chemotherapy alone. Each received potent agents that may attenuate the body’s normal inflammatory signaling, thereby obscuring typical clinical warning signs. Corticosteroids reduce lymphoid tissue, weaken immune cell function, and lower pro-inflammatory cytokines, which can mask typical signs such as abdominal irritation and fever [6,7,16,17]. Each patient also received targeted biologic agents: ruxolitinib (JAK1/2 inhibitor), anakinra (IL-1 receptor antagonist), tocilizumab (IL-6 receptor antagonist), and/or eculizumab (C5 inhibitor). These agents block specific inflammatory pathways that normally help the body respond to infection and injury [18,19,20,21,22,23,24]. As a result, common signs of a perforated gut, such as fever, leukocytosis, tachycardia, and elevated lactate, were absent, which can be misleading. Unlike classic neutropenic perforation, where fever and pain are often still present, these cases showed a markedly suppressed inflammatory response [5,6].

### 3.3. Rethinking Risk Factors and Diagnostic Triggers

These cases indicate that traditional risk markers need to be reevaluated. Severe neutropenia was not consistently present at the time of perforation. Relying solely on vital signs or laboratory results is insufficient. Instead, a constellation of subtle clinical findings may serve as early indicators:

- Ongoing or unexplained gastrointestinal symptoms: persistent bleeding (Case 1), feeding intolerance without progress (Cases 2 & 3), and persistent abdominal distension (Cases 2 & 3).
- Chronic, smoldering bowel issues: pre-existing conditions such as neutropenic enterocolitis or pneumatosis intestinalis may create an environment for contained, slow-developing perforation [6,7].
- Incidental imaging clues: Retrospective analysis identified free air in two cases, underscoring that radiographic “soft signs” must be taken seriously in this context.

**Figure 5:**
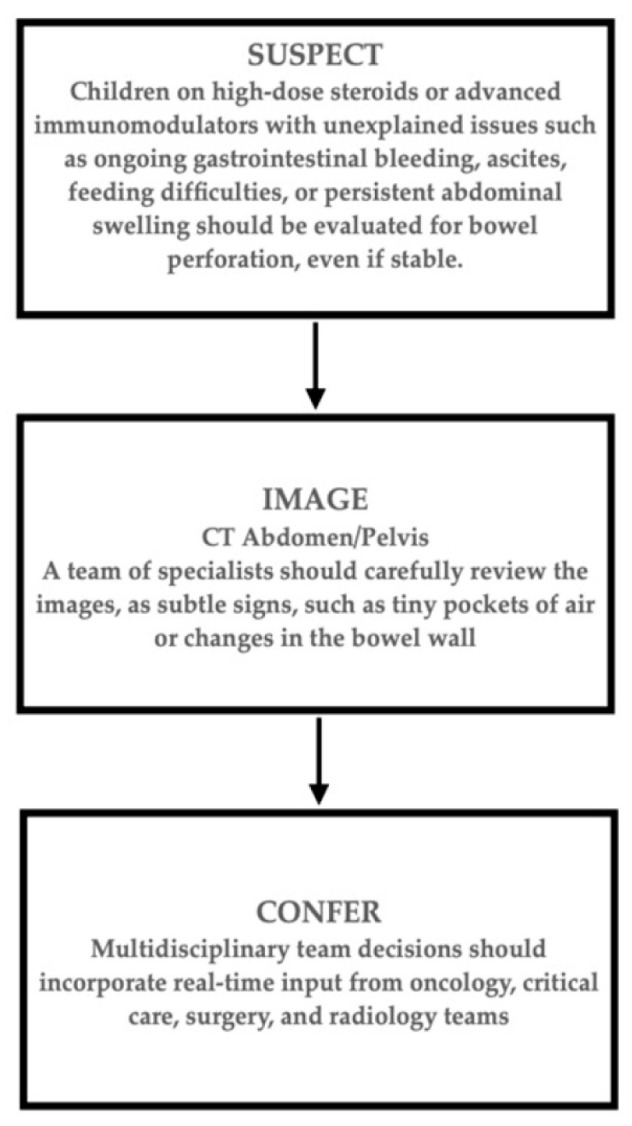
Monitoring high-risk immunocompromised children for silent intestinal perforation.

These challenges underscore a significant gap in monitoring high-risk immunocompromised pediatric patients. Delaying intervention until classic signs of peritonitis emerge may result in adverse outcomes. A proactive, multi-step approach is warranted. We suggest the following clinical steps (see Figure 5):

1. Increased Clinical Suspicion: For any child on high-dose steroids or advanced immunomodulators (such as JAK inhibitors or cytokine blockers) with unexplained problems, including ongoing gastrointestinal bleeding, ascites, feeding difficulties, or persistent abdominal swelling, doctors should consider bowel perforation, even if the child appears stable.
2. Quick and Thorough Imaging: Be ready to order detailed abdominal imaging (such as a CT scan) for any concerning gastrointestinal symptom. A team of specialists should carefully review the images, as subtle signs, such as tiny pockets of air or changes in the bowel wall, can be missed.
3. Team-Based Decisions: Treatment decisions should incorporate real-time input from oncology, critical care, surgery, and radiology teams. It is important to clearly share details of the patient’s immunosuppressive medications so that everyone can correctly interpret the clinical and imaging findings.

### 3.4. Limitations

Our manuscript has limitations inherent in its nature as a small, retrospective case series. The infrequent occurrence of this complication limits the feasibility of conducting extensive studies within a single institution, and it remains unclear whether specific immunomodulators are directly correlated with silent perforations. Furthermore, the severity of patients’ critical conditions and sedation may obscure abdominal symptoms. Nonetheless, the consistent findings across all three cases underscore a significant clinical oversight. Potential biases include selection bias, institution-specific diagnostic and imaging practices, and the lack of standardized imaging protocols.

## 4. Conclusions

Progress in pediatric hematology–oncology has improved survival but has also altered the types of serious complications observed. This series highlights a key diagnostic challenge: intestinal perforation without the usual signs of peritonitis. In children receiving modern, multi-drug immunosuppression, typical symptoms of bowel emergencies are often absent because these drugs mask them. As a result, doctors cannot rely solely on clinical stability to assess gut health.

To optimize safety for vulnerable pediatric patients, clinicians should adopt a proactive approach, recognizing that subtle gastrointestinal symptoms or minor imaging abnormalities may be the earliest signs of bowel perforation. Diagnostic strategies must account for the potential of immunomodulatory therapies to obscure typical inflammatory responses. Improved outcomes depend on early identification of these atypical cases, enabling surgical intervention before severe sepsis develops. This report underscores the necessity for pediatric oncology, immunology, and critical care teams to remain vigilant for this concealed risk, even in the absence of classic clinical signs.

## Data Availability

The original contributions presented in this study are included in the article. Further inquiries can be directed to the corresponding author.

## Abbreviations

The following abbreviations are used in this manuscript:

HSCT	Hematopoietic stem cell transplant
GVHD	Graft-versus-host disease
TMA	Thrombotic microangiopathy
CT	Computed tomography
HLH	Hemophagocytic lymphohistiocytosis
MRSA	Methicillin-resistant staphylococcus aureus
